# Pharmacogenetics of Lethal Opioid Overdose: Review of Current Evidence and Preliminary Results from a Pilot Study

**DOI:** 10.3390/jpm13060918

**Published:** 2023-05-30

**Authors:** Leen Magarbeh, Ilona Gorbovskaya, Richard Wells, Reuven Jhirad, Bernard Le Foll, Daniel J. Müller

**Affiliations:** 1 Department of Pharmacology and Toxicology, Faculty of Medicine, University of Toronto, Toronto, ON M5S 1A8, Canada; leen.magarbeh@camh.ca (L.M.); bernard.lefoll@camh.ca (B.L.F.); 2 Centre for Addiction and Mental Health, Toronto, ON M5T 1R8, Canada; ilona.gorbovskaya@camh.ca; 3 Factor-Inwentash Faculty of Social Work, University of Toronto, Toronto, ON M5S 1V4, Canada; 4 Office of the Chief Coroner and Ontario Forensic Pathology Service, Toronto, ON M3M 0B1, Canada; richard.wells@ontario.ca (R.W.); reuven.jhirad@ontario.ca (R.J.); 5 Institute of Medical Sciences, University of Toronto, Toronto, ON M5S 1A8, Canada; 6 Department of Psychiatry, University of Toronto, Toronto, ON M5T 1R8, Canada; 7 Department of Family and Community Medicine, Faculty of Medicine, University of Toronto, Toronto, ON M5G 1V7, Canada; 8 Translational Addiction Research Laboratory, Campbell Family Mental Health Research Institute, Centre for Addiction and Mental Health, Toronto, ON M5T 1R8, Canada; 9 Acute Care Program, Centre for Addiction and Mental Health, Toronto, ON M5T 1R8, Canada; 10 Dalla Lana School of Public Health, University of Toronto, Toronto, ON M5T 3M7, Canada; 11 Waypoint Research Institute, Waypoint Centre for Mental Health Care, Penetanguishene, ON L9M 1G3, Canada

**Keywords:** pharmacogenetics, post-mortem, opioids, overdose, CYP450

## Abstract

There has been a worldwide substantial increase in accidental opioid-overdose deaths. The aim of this review, along with preliminary results from our pilot study, is to highlight the use of pharmacogenetics as a tool to predict causes of accidental opioid-overdose death. For this review, a systematic literature search of PubMed^®^ between the time period of January 2000 to March 2023 was carried out. We included study cohorts, case–controls, or case reports that investigated the frequency of genetic variants in opioid-related post-mortem samples and the association between these variants and opioid plasma concentrations. A total of 18 studies were included in our systematic review. The systematic review provides evidence of the use of *CYP2D6*, and to a lower extent, *CYP2B6* and *CYP3A4/5* genotyping in identifying unexpectedly high or low opioid and metabolite blood concentrations from post-mortem samples. Our own pilot study provides support for an enrichment of the CYP2B6*4-allele in our methadone-overdose sample (*n* = 41) compared to the anticipated frequency in the general population. The results from our systematic review and the pilot study highlight the potential of pharmacogenetics in determining vulnerability to overdose of opioids.

## 1. Introduction

Opioids are commonly prescribed as pain medication but are also abused as illicit drugs. Opioid-related mortality caused by adverse drug reactions and unintentional overdose is a serious and global health concern. According to provisional data from the National Centre for Health Statistics at the Centre for Disease Control and Prevention (CDC), there were more than 100,000 drug overdose deaths in the United States in 2021 alone, including illicit and prescription opioids, which is a nearly 17% increase in opioid-related mortality compared to the same period in 2020 [1]. In Canada, recent statistics show that the apparent opioid-related mortality rate increased by 91% over the two years of the COVID-19 pandemic (from April 2020 to March 2022) compared with previous years (2018–2019) [2]. Specifically, between January and June 2022, there were at least 3500 apparent opioid-related deaths in Canada, of which 97% were accidental. The increased availability of potent synthetic opioids, mainly fentanyl and fentanyl analogs, contributed to the increased overdose fatalities and were involved in nearly 75% of those deaths [2]. Furthermore, opioid-related deaths involved multiple drugs, including psychostimulants (e.g., cocaine, amphetamine), benzodiazepine and alcohol, highlighting the polysubstance nature of the opioid crisis [1,2].

In Canada, opioid prescribing for pain management is strongly regulated and controlled and their use is legal only when they are prescribed by licensed practitioners [3]. In 2018, almost one in eight people in Canada were prescribed an opioid, mainly codeine, hydromorphone, morphine, oxycodone, or fentanyl [3]. While the dispensing of opioids is strictly regulated, the increased demand for pain relief medications has led to the widespread redistribution or re-selling of prescription opioids (i.e., prescription diversion) via illicit street markets [3]. In a study conducted on the patterns of opioid prescribing in Canada, it was revealed that 37% of opioid-dependent individuals received their opioids solely from a licensed physician, while 21% received their opioids from the illicit street markets [4]. Furthermore, prescription opioids not marketed in Canada can be diverted illegally into the country [3]. This increased availability of non-prescription or illicit opioids has contributed to the rise in accidental overdose deaths [2].

The long-term administration of opioids should be medically supervised as their chronic use can lead to the development of physiological dependence, addictive behavior and misuse [5]. Opioids work by activating one or more of the several opioid receptors (mu, kappa and delta) and the nociceptin peptide receptors in the brain [6]. In the early stages of use, opioids stimulate the mesolimbic (midbrain) reward system. The compulsion for continued opioid use is related to the development of tolerance (the need to take higher and higher doses of opioids to achieve the same reward) and dependence (susceptibility to withdrawal symptoms) [7]. Lethal overdose with opioids occurs through excessive activation of the mu-opioid receptors in the locus coeruleus neurons in the brain, resulting in central nervous system (CNS) depression, drowsiness, suppressed respiration and a severe drop in blood pressure [5,6]. 

The considerable rise in opioid-related fatalities has prompted an increase in public health interventions aimed at curtailing the impact of prescription opioid analgesics on the current overdose epidemic. These interventions involve opioid prescribing, monitoring and tapering guidelines for healthcare providers, in addition to educational courses for patients that describe the risks and misuse associated with opioid analgesics [8]. Currently, there are limited proposed biological strategies available to address the global opioid overdose problem [9]. Strategies that target genetic and epigenetic factors may accelerate the development of effective interventions. For example, recent studies showed that there is genetic vulnerability to the development of substance abuse [10] and a genetic risk score composed of single nucleotide polymorphisms (SNPs) can be used to predict the risk of opioid addiction [9]. Therefore, there is a need for pharmacogenetic-based strategies to predict which individuals are at a greater risk of unintended lethal adverse effects from opioids. 

Pharmacogenetics describes how genetic variations affecting drug pharmacokinetics (i.e., metabolism or transport) or pharmacodynamics (i.e., receptors) may contribute to the interindividual differences in response, tolerance and adverse effects to medications [11]. The pharmacogenetics of opioids has been extensively described in clinical studies including individuals receiving pain management therapies to maximize therapeutic effects, improve treatment outcomes and minimize toxicity [12,13,14]. According to the Clinical Pharmacogenetics Implementation Consortium (CPIC), polymorphisms in CYP2D6, a drug-metabolizing enzyme, have been clearly associated with large interindividual variations in codeine and tramadol response—ranging from poor analgesia to life-threatening CNS depression at standard doses [15]. Therefore, prescribing guidelines recommending pharmacogenetic testing prior to the selection and dosing of clinically relevant opioids have been published [15]. In addition, polymorphisms in other opioid metabolizing enzymes (i.e., CYP2B6, CYP3A4), in the opioid receptors (*OPRM1*), and the opioid transporters (*ABCB1*), have shown significant associations with variability in opioid dosing requirements, efficacy and adverse effects [13]. The majority of those associations were based on clinical prospective or retrospective studies or case reports. Limited research has been done on the pharmacogenetics of opioid overdose using post-mortem data.

Pharmacogenetics may be helpful in the field of post-mortem toxicology, as it can be used to definitively identify deaths related to suicide, accidents and unknown causes. Retrospective analysis of post-mortem cases revealed that polymorphisms in CYP enzymes, which metabolize selected opioids including codeine, tramadol, methadone and fentanyl, correlate with the serum level of opioids and their metabolites and may serve as an adjunct in certifying the cause of death in unexpected high or low metabolite/parent drug ratios [16,17,18]. As such, the purpose of this review is to summarize evidence of opioid pharmacogenetics in post-mortem cases to highlight the importance of using pharmacogenetics as a tool to identify causes of accidental opioid-overdose deaths. Furthermore, this brief review also provides preliminary results from our pilot study on the association of genetic variation and opioid overdose in post-mortem cases investigated by the Office of the Chief Coroner of Ontario, Canada.

## 2. Materials and Methods

### 2.1. Systematic Review

#### 2.1.1. Identification of Data through Public Databases and Registers

A systematic literature search of published articles was conducted using PubMed, from January 2000 to March 2023. The following keywords were used: (pharmacogenetics OR variants OR polymorphisms OR SNPs) AND (opioids OR *NameOfTheDrug*) AND (post-mortem OR deaths OR fatalities). Bibliographies of included research articles were hand-searched for additional references not identified in our primary searches. This systematic review followed the 2020 PRISMA (Preferred Reporting Items for Systematic Reviews and Meta-Analyses) reporting recommendations. 

#### 2.1.2. Data Selection

Articles were included if they were: (1) cohort studies, case–control, or case reports, (2) published in English between January 2000 and March 2023, (3) investigated gene variants in post-mortem studies where opioids were the cause of death.

#### 2.1.3. Data Extraction and Quality Assessment

All articles identified by the search strategy were assessed for eligibility independently by both reviewers (LM and IG). Information extracted from each eligible article included: (1) author names, study design and publication year; (2) sample size; (3) case characteristics (i.e., age, sex ethnicity/ancestry) (4) name of opioid investigated; (5) phenotype assessed; (6) genes and SNPs assessed; and (7) main findings of the study. An assessment of study quality was conducted independently by two reviewers (LM and IG).

### 2.2. Pilot Study

#### 2.2.1. Recruitment

This study was approved by the Office of the Chief Coroner of Ontario (OCC) and the Research Ethics Board of the Centre of Addiction and Mental Health (CAMH) in Toronto, Canada. Accidental opioid-related fatalities were identified through our collaboration with the Regional Supervising Coroner, RW, and Deputy Chief Coroner, RJ, from the OCC and Ontario Forensic Pathology Service (OFPS). In accordance with Ontario’s Coroners Act, all deaths that are sudden, unexpected and/or unnatural must be reported to the OCC. The coroner classifies the manner of death according to five categories: natural, accident, suicide, homicide and undetermined. The investigating coroner ascertains the cause and manner of death according to data collected in the course of the investigation, which may include autopsy, post-mortem examination and detailed toxicological testing and chemical analysis.

A post-mortem chemical examination usually includes detailed toxicological testing for drugs by immunoassay and gas chromatography–mass spectrometry (GC–MS), and screening for volatiles by headspace GC. This is followed by confirmation and quantitation by GC–MS or liquid chromatography (LC)–MS/MS, as required. Deaths related to opioid overdose were identified by the OCC based on a toxicological analysis that revealed (1) an opioid concentration sufficiently high (above the fatal reference range) to cause death, or (2) a combination of drugs, including at least one opioid present at a high concentration and other intoxicants, such as CNS stimulants, benzodiazepines, or alcohol. Deaths were not considered related to opioid use if another drug was present at a high enough concentration to cause death. Deaths in which other circumstantial factors could have on their own resulted in death (i.e., suicide, homicide, external injuries, motor vehicle collisions, and disease) were not included. 

Based on the above criteria, 119 accidental opioid overdose cases (78 from 2021–2023 and 41 from 2013–2014) were included in this pilot study. The data collected in this study were coded and analyzed anonymously. No personal identifiers were collected.

#### 2.2.2. Blood Sample Collection and Genotyping

A total of 41 blood samples (methadone-overdose cases only) obtained from the OCC/OFPS were sent for DNA extraction and genotyping at the CAMH Biobank and Molecular Core Facility (Centre for Addiction and Mental Health, Toronto, ON, Canada). Genomic DNA was extracted from blood samples using a modified version of the FlexiGene DNA kit (QIAGEN, Hilden, Germany). Genotyping was performed using standard TaqMan^®^ Assays (Thermo Fisher Scientific, Waltham, MA, USA) according to the manufacturer’s protocol. Two SNPs were genotyped in the *CYP2B6* gene, rs3745274 (*9, C516G/T) and rs2279343 (*4, A785G) and one SNP in the *OPRM1* gene, rs1799971 (A118G). SNP minor allele and genotype frequencies were determined. Genotyping results were reviewed by two laboratory staff blind to the clinical data. Ten percent of the sample was re-genotyped for quality control.

#### 2.2.3. Statistical Analysis

All analyses were conducted using R Version 4.0.4. (R Foundation for Statistical Computing Platform, 2021) and RStudio Version 1.4.1106 (RStudio Inc., Boston, MA, USA, 2021). Descriptive statistics for demographic and clinical characteristics were generated using the Fisher exact test for categorical variables and the Kruskal–Wallis test for continuous variables.

## 3. Results

### 3.1. Systematic Review

**Figure 1:**
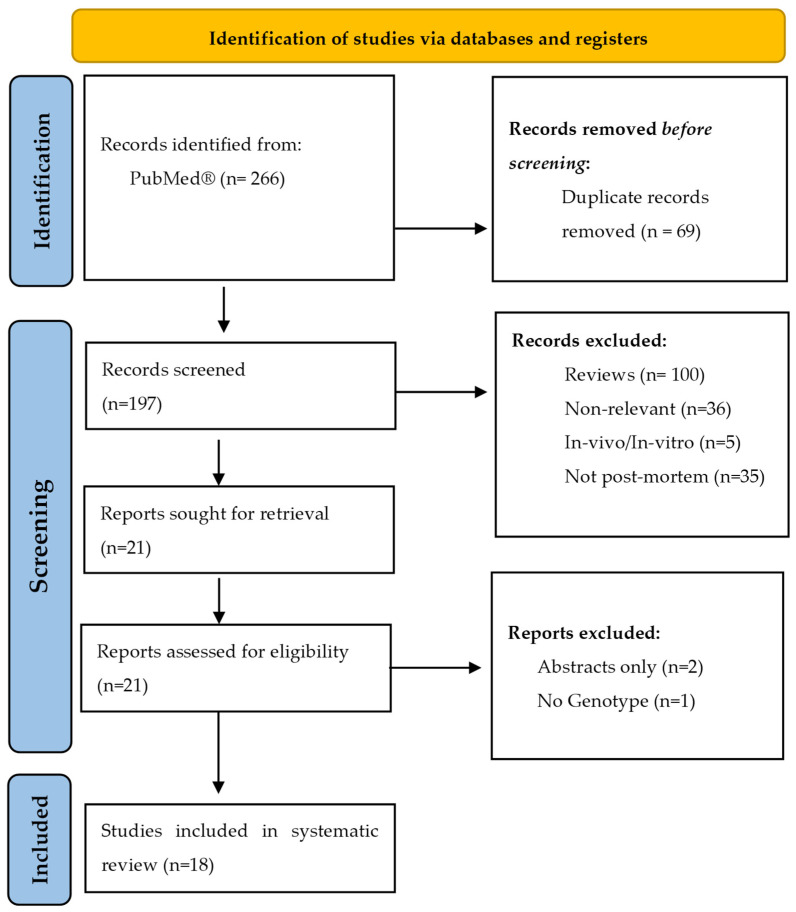
PRISMA flow diagram.

The systematic search produced a total of 266 articles. A summary of the article selection process is presented in the PRISMA flow diagram (Figure 1). After title, abstract and full-text screening, a total of 18 articles were eligible for inclusion in this systematic review. The characteristics of available reported data from each article are presented in Table 1.

Briefly, more than 66% (*n* = 12) of the included studies were retrospective cohorts in which forensic autopsy cases were reviewed and gene-variant frequencies were tested for an association with the opioid and respective metabolite concentrations. Only six studies were case–controls in which the frequencies of gene variants in opioid-related fatality cases were compared to the frequencies in control samples (which were identified as either healthy volunteers, individuals with opioid addiction, fatalities caused by suicide, or drugs other than opioids). The commonly investigated opioids were methadone (*n* = 7), codeine (*n* = 3), tramadol (*n* = 3), oxycodone (*n* = 3), hydrocodone (*n* = 1), morphine (*n* = 1), and fentanyl (*n* = 1). The most investigated gene was *CYP2D6*, followed by *CYP2B6*, *OPRM1*, *ABCB1*, *CYP3A4/5* and *COMT*.

#### 3.1.1. CYP2D6 and Opioids

**Figure 2:**
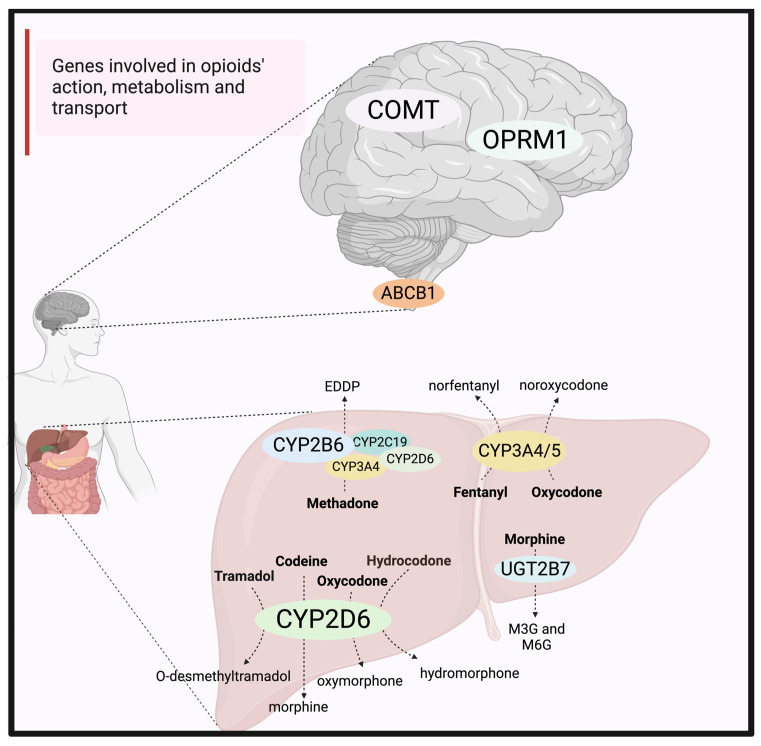
Genes involved in opioids’ action, metabolism, and transport. CYP2D6 metabolizes tramadol, codeine, hydrocodone and oxycodone to their more active metabolites, O-desmethyltramadol, morphine, hydromorphone and oxymorphone, respectively. Morphine is further metabolized by UGT2B7 to M3G and M6G (which has pharmacological activity). Fentanyl is metabolized by both CYP3A4 and CYP3A5 to an inactive metabolite, norfentanyl. Methadone is mainly metabolized by CYP2B6, with little contribution from CYP3A4, CYP2D6, or CYP2C19, to form an inactive metabolite, EDDP. Several opioids are substrates of the P-glycoprotein transporter, encoded by the *ABCB1* gene, which is located in various locations in the body, including the liver and intestine and at the blood–brain barrier. In the brain, opioids bind and activate the mu-opioid receptor encoded by the *OPRM1* gene. The C*OMT* gene may also affect the opioid’s action. ABCB1 = P-glycoprotein encoding gene; COMT = catechol-o-methyltransferase encoding gene; DRD2 = dopamine receptor D2 subtype encoding gene; EDDP = 2-ethylidene-1, 5-dimethyl-3, 3-diphenylpyrrolidine; M3G = morphine-3-glucuronide; M6G = morphine-6-glucuronide; OCT1 = organic cation transporter 1 encoding gene; OPRM1 = mu-opioid receptor encoding gene; UGT2B7 = UDP-glucuronosyltransferase 2B7.

CYP2D6 is a drug-metabolizing enzyme involved in the metabolism of approximately 20% of clinically used drugs, including some important opioids (see Figure 2) [36]. The CYP2D6-encoding gene is highly polymorphic, with over 130 identified variants. The combination of *CYP2D6* genetic variants constitutes four different phenotypic subgroups based on the rate of drug metabolism. These four phenotypic groups are ultra-rapid metabolizers (UM), normal metabolizers (NM) or formerly extensive metabolizers (EM), poor metabolizers (PM) and intermediate metabolizers (IM) [36]. A recent retrospective cohort including 75 US military veteran deaths has found that approximately 7% of individuals who died due to an opioid overdose carried a UM phenotype [32]. 

CYP2D6 metabolizes codeine, tramadol and oxycodone into their more pharmacologically active metabolites, which are morphine, O-desmethyltramadol and oxymorphone, respectively (Figure 2) [13]. Tramadol, which is a synthetic opioid, was commonly investigated in post-mortem fatalities. This is because tramadol concentrations have shown a consistent correlation with the *CYP2D6* metabolizer phenotypes in clinical investigations [15]. In this review, several studies examined the association between *CYP2D6* metabolizer phenotypes and tramadol post-mortem blood concentrations. These studies concluded that *CYP2D6* genotyping may be important in identifying the cause of unexpectedly high or low tramadol-metabolite ratios in post-mortem blood samples [19,27]. Furthermore, one study looked at 16 variants from five genes involved in tramadol pharmacokinetics and pharmacodynamics and concluded that a set of 16 loci from these five genes can predict tramadol/metabolite ratio with over 90% accuracy, which is greater than using *CYP2D6* alone [30]. Similarly, the oxycodone-to-oxymorphone concentration ratio showed a significant correlation with *CYP2D6* activity when death was unrelated to intoxication and *CYP2D6* PMs and IMs had significantly higher oxycodone concentrations compared to EMs and UMs [35].

In contrast, for codeine, studies concluded that there was a large variability in the calculated post-mortem concentration ratios of codeine to its metabolite, morphine, which was not explained by the *CYP2D6* genotypes alone [16,24,28]. A recent systematic review has shown that the reliability and validity of measuring morphine concentrations in post-mortem samples are low [37]. This is because there are post-mortem changes, including post-mortem morphine metabolism and redistribution, that could result in a wide range of morphine blood concentrations reported in deaths [37]. Morphine is mainly metabolized into morphine-3-glucuronide (M3G, inactive metabolite) and morphine-6-glucuronide (M6G, equal or greater affinity at the mu-opioid receptor than morphine) by the UDP-glucuronosyltransferase 2B7 (UGT2B7) enzyme (Figure 2). However, there is reported evidence of post-mortem of de-glucuronidation of M3G and M6G back to morphine by bacterial beta-glucuronidase, or spontaneously [37]. Moreover, drugs with a high volume of distribution and high lipophilicity such as morphine are quickly distributed to tissues. After death, these drugs are released into plasma, resulting in a post-mortem increase in concentrations, a phenomenon known as post-mortem redistribution [37]. Therefore, when interpreting post-mortem codeine findings, analysis of morphine and its glucuronide metabolites should be considered.

In summary, evidence suggests a correlation between *CYP2D6* genotypes and opioid blood concentration in forensic cases of tramadol and oxycodone toxicity. As for codeine, the relevance of *CYP2D6* genotyping in the determination of unexpected codeine/metabolite post-mortem blood concentrations has still to be shown.

#### 3.1.2. CYP2B6 and Methadone

CYP2B6, which mediates the metabolism of methadone, is also a highly polymorphic gene, with more than 30 variant alleles identified [38]. The CYP2B6*4 is an increased function allele, while the *9-allele produces an enzyme with decreased activity. The CYP2B6*4 allele is usually present along with the *9-allele to form the CYP2B6*6 haplotype, which is the most common and clinically significant haplotype that results in reduced CYP2B6 hepatic expression and activity [38]. CYP2B6 is the main enzyme involved in the metabolism of methadone to an inactive metabolite 2-ethylidene-1,5-dimethyl-3,3-diphenylpyrrolidine (EDDP) (Figure 2) [13].

Methadone has been the most investigated opioid in post-mortem cases. Methadone is a widely used medication in opioid replacement therapy with the goal of transitioning opioid-use disorder individuals from an abused faster-acting opioid to a clinically controlled slower-acting opioid and attenuating the occurrence of opioid withdrawal, craving and opioid-seeking behaviors [39]. Methadone is an opioid agonist, with the (R)-methadone having a 10-fold higher affinity to the opioid receptors (mu, delta and kappa) compared to the (S)-methadone [13]. Seven studies examined the frequencies of several gene variants in methadone-related deaths and their association with methadone or EDDP plasma concentrations. In one retrospective cohort including 40 cases of Caucasian ethnicity, CYP2B6*4, *9 and *6-alleles were associated with higher post-mortem methadone concentration [21]. In two case–control studies, CYP2B6*4 and *9 and *6-allele frequencies were enriched in methadone-related deaths, compared to the frequencies in deaths caused by drugs other than opioids [29], or the frequency in healthy volunteers [23].

In summary, *CYP2B6* genotypes, mainly the CYP2B6*6 haplotype, have been linked with increased susceptibility to unintentional methadone fatality [21]. Because CYP2B6*6 is the most clinically significant haplotype, future post-mortem investigation should examine the CYP2B6*6 haplotype in methadone-related overdose deaths. 

#### 3.1.3. CYP3A4/5 and Opioids

CYP3A4 is the most abundant cytochrome enzyme in the liver. Numerous SNPs have been identified in the CYP3A4-encoding gene, however, most of the exonic SNPs have a minor allele frequency of less than 5% in the majority of populations and the impact of genetic variations appears to be relatively modest compared to *CYP2D6* poor metabolizers. One commonly investigated variant, the CYP3A4*1B (rs2740574), has been associated with a lower CYP3A4 enzymatic activity [25]. In contrast to *CYP3A4,* the presence of a non-functional *CYP3A5* (CYP3A5*3, *6, or *7) is the norm in many populations and it is present in 80–85% of Europeans [40]. CYP3A4 and CYP3A5 share common substrates, and both enzymes are involved in the metabolism of fentanyl into an inactive metabolite, norfentanyl (Figure 2) [13].

Fentanyl is a synthetic opioid and overdose with fentanyl is most likely due to illicit non-prescription use, notably, now that the fentanyl or fentanyl derivatives are often present in the illicit drug supply. Fentanyl has high potency—it is 100 times more potent than morphine—because of its high lipophilicity [41]. This greater potency significantly lowers the threshold for the risk of overdose and death [41]. Only one study examined gene variants in *CYP3A4* and *CYP3A5* and fentanyl-related deaths [18]. With a small sample size (*n* = 25), the study found a nominal association between CYP3A4*IB and CYP3A5*3 gene variants and fentanyl/norfentanyl ratios, where homozygous CYP3A5*3 individuals showed impaired metabolism of fentanyl if they additionally carried the CYP3A4*1B variant, especially the homozygous genotype [18]. The study concluded that these genes may serve as an adjunct in certifying fentanyl toxicity in post-mortem cases. 

CYP3A4 is also involved in the metabolism of methadone. One study involving 136 accidental methadone-only fatalities demonstrated a correlation between polymorphisms of the *CYP3A4* gene and increased likelihood of accidental fetal methadone intoxication and showed significant enrichment of the CYP3A4*1B allele in the post-mortem cases, compared with the general population [25].

In summary, *CYP3A4/5* variants are highly present in the majority of populations but with a low frequency. Therefore, larger studies are needed to accurately assess the correlation of *CYP3A4* and *CYP3A5* gene variants with impaired opioid metabolism and accidental opioid toxicity.

#### 3.1.4. ABCB1, OPRM1, COMT and Opioids

The *ABCB1* gene encodes a P-glycoprotein efflux transporter, located in the liver and intestine and at the blood–brain barrier (BBB) [42]. The P-glycoprotein protein pump at the BBB regulates the concentration of certain opioids in the brain (e.g., methadone, morphine) [42,43]. Common polymorphisms in the *ABCB1* gene, including rs1045642 (C3435T), rs2032582 (G2677T/A) and rs1128503 (C1236T), have shown an association with decreased P-gp expression and/or function [42]. These gene variants were investigated in studies including methadone- and codeine-related deaths. The studies reported that individuals who carried the 1236T variant had statistically lower morphine (codeine’s metabolite) blood concentrations than wild-type carriers [24] and individuals with a 3435T genotype had higher methadone brain/blood concentration ratios [34]. These results indicate that *ABCB1* genetic variants, which alter P-gp expression or function, may play a role in determining active opioid concentrations reaching their site of action in the brain.

The *OPRM1* gene encodes the mu-opioid receptor, which is the main site of action for all opioids. The most commonly studied variant in the *OPRM1* gene is the A118G SNP, which results in reduced protein expression and reduced signal transduction pathways [13]. The A118G SNP has been consistently associated with increased morphine dosing requirements and has been linked with susceptibility to drug addictions [44,45]. The A118G SNP has also been shown to reduce the analgesic effects of opioids, providing a rationale for dose escalations [37]. For morphine, the A118G SNP was shown to have a protective effect against respiratory depression mainly caused by morphine’s metabolite, M6G, in several case reports [37]. In this review, one study showed a higher prevalence of the A118G variant in a population of healthy volunteers (*n* = 100), compared with 84 post-mortem methadone-related fatalities [23], while another study reported no significant difference in the frequency of this variant in deceased individuals with opioid addiction (*n* = 274) compared to individuals living with opioid-use disorder (*n* = 309) [26]. Furthermore, one study has demonstrated that the 118G variant was associated with higher benzodiazepine concentrations when it is present as a co-intoxicant in methadone-related fatalities, but not with methadone or EDDP concentrations [21].

Other genes are known to be involved in the development of addiction and response to opioids, such as the Catechol-o-methyltransferase-encoding gene, *COMT*. The COMT enzyme affects opioids’ action via modulation of the dopamine–enkephalin pathway [13]. A common polymorphism in the COMT-encoding gene is the Val158Met (rs4680) [13]. The presence of this polymorphism leads to a three-to-four-fold decrease in enzyme activity and several studies involving postoperative pain or cancer cohorts reported lower morphine dosing requirements in individuals carrying the 158Met variant [13]. While polymorphisms in the COMT-encoding gene were not as commonly investigated in post-mortem fatalities, one study reported a significantly lower frequency of the 158Met variant in methadone- and morphine-related deaths and concluded that there is a possible association between the presence of the Val158Met variant and reduced risk of death [26].

In summary, assessing *ABCB1*, *OPRM1* and *COMT* polymorphisms in opioid-related deaths needs further evaluation, especially when morphine and/or methadone are involved.

#### 3.1.5. Other Interactions with Opioids

This present analysis of opioid-related fatalities revealed that the majority of opioid-related deaths involved mixed intoxications by CNS depressants (i.e., benzodiazepines, hypnotics and/or alcohol), antidepressants, or other co-medications. In a study that examined 68 codeine-related deaths (CRDs), the presence of CNS depressants was significantly associated with lower codeine concentration in CRDs compared to CRDs in which CNS depressants were not detected [24]. This indicates that the presence of co-intoxicants may lead to toxicity at lower opioid doses. Furthermore, in another study involving 174 oxycodone-related deaths, the CYP2D6 metabolizer phenotypes correlated with the concentration of oxymorphone/oxycodone ratio when death was unrelated to intoxications by benzodiazepines, alcohols, or other opioids [35]. In summary, when analyzing opioid/metabolite concentrations in post-mortem samples, the presence of co-intoxicants should be considered.

### 3.2. Pilot Study Results

Demographic data for the 119 cases are outlined in Table 2. The majority of cases were male (65.5% (*n* = 78/119)). Fentanyl and fentanyl analogs (carfentanil) were involved in 60.5% of opioid-related deaths (*n* = 72), followed by methadone, oxycodone, morphine, hydromorphone and heroin. Co-intoxicants with a CNS stimulant, mainly amphetamine, methamphetamine and cocaine, were present in 82.4% of cases. Co-intoxicants with alcohols, benzodiazepines, or antidepressants were present in 25.2%, 8.4% and 5.0% of accidental deaths, respectively.

For the cases involving methadone-related deaths (*n* = 41), 25 samples have been genotyped for the CYP2B6*4, CYP2B6*9 variants and 41 samples for the *OPRM1* A118G variant. Table 3 shows the observed genotype and minor allele frequency for the CYP2B6*4 and CYP2B6*9, respectively. Our initial results suggested a minor allele frequency of 0.3 for the *CYP2B6* *9 variant, which is consistent with the reported global allele frequency (www.ensembl.org). Notably, we detected a difference in the distribution of the CYP2B6*9 between males and females in our sample (*p*-value = 0.007) (Table 3). As for CYP2B6*4, we showed enrichment of this minor allele (0.3) compared to the reported global allele frequency (0.13) (www.ensembl.org). With respect to the *OPRM1* A118G gene variant, we observed a minor allele frequency of 0.05 in our sample of methadone overdose (Table 3).

## 4. Discussion

Accidental death due to opioid overdose is a major public health concern worldwide. The results of this systematic review have shown that methadone, codeine, tramadol, oxycodone and fentanyl were the most commonly investigated opioids in post-mortem cases. According to the CDC, drug overdose deaths involving prescription opioids (including methadone) in the US increased from 3442 in 1999 to 17,029 in 2017 [1]. From 2017 to 2021, this number decreased to 16,706 reported deaths [1]. In contrast, synthetic opioids other than methadone (primarily fentanyl) were the main driver of drug overdose deaths with a nearly 7.5-fold increase from 2015 to 2021 [1], which explains the majority of fentanyl-involved deaths in our cases collected more recently, i.e., from 2021 to 2023. 

In this study, we underscore the importance of how pharmacogenetics can be used in interpreting the cause and manner of opioid-related deaths. This is mainly because gene variants of enzymes that metabolize or transport opioids change the bioavailability and the therapeutic/toxic dose of the drugs. Specifically, the results of the systematic review showed that gene variants in *CYP2D6* corresponding to the different metabolizer phenotypes were strongly correlated with differences in the O-desmethyltramadol/tramadol concentration ratios in post-mortem samples [19,27,35]. To a lower extent, gene variants in the *CYP2B6* and the *CYP3A4/5* showed a correlation with the methadone and fentanyl concentrations, respectively [18,21]. In our pilot study, the *4-variant in the *CYP2B6* gene was enriched in our methadone-overdose cases compared to the global reported variant frequency. Similarly, selected variants in the *CYP2B6* and *CYP3A4* genes were enriched in post-mortem opioid-overdose cases, compared to a control population [25,29]. Therefore, *CYP2D6*, *CYP2B6* and *CYP3A4* genotyping may be used as a supplementary tool to certify opioid toxicity and to interpret unexpected post-mortem opioid and metabolite concentrations. More research analyzing variants in the *ABCB1*, *ORPM1* and *COMT* genes, especially in methadone- and morphine-related deaths, is needed.

The use of a single-gene approach (i.e., targeted single-nucleotide polymorphisms in one gene) to explain variations in post-mortem opioid concentrations has been employed in the majority of the included studies. For example, the investigation of the SNP, A118G, in the *OPRM1* gene did not show significant associations with opioid or metabolite concentrations in post-mortem cases [21,24,26]. However, a pathway-driven predictive model including genes that are involved in the absorption, distribution, metabolism, excretion and response of opioids may be more useful in predicting the opioid/metabolite concentrations in post-mortem samples [30]. For example, Wendt et al. 2019, reported that a pathway-driven model using five genes (*CYP2D6*, *UGT2B7*, *ABCB1*, *OPRM1*, *COMT*) predicted the tramadol and metabolite concentrations in 208 post-mortem cases with over 90% accuracy compared to using one gene alone [30]. Furthermore, a shift from a single-gene approach to using a genome-wide genotyping approach (i.e., genome-wide association studies or GWAS) in identifying opioid response and toxicity is warranted. One study (*n* = 37), which used a genome-wide screen approach, identified five additional single nucleotide polymorphisms that were associated with decreased metabolite/tramadol ratios in post-mortem cases [33]. An alternative aggregated genome-wide approach, such as polygenic risk scores (PRS), which is a single variable that predicts the risk to a given trait by considering the additive effects of common variants across the human genome, should be considered. To date, it has only been used in one study related to opioid misuse [46]. Currently, a PRS for the prediction of lethal opioid overdose has not been developed. 

The potential role of pharmacogenetics in accidental fatal opioid overdose cases remains complex, as it can be confounded by other factors such as post-mortem drug redistribution, the presence of co-intoxications, co-medication, the risk of phenoconversion and the variation of allele frequencies across populations of different ancestries. Post-mortem tissue redistribution of opioids, especially those with a high volume of distribution or high lipophilicity, may lead to artifactually decreased or increased opioid blood concentrations at the time of sample collection [28,41,47]. However, peripheral or femoral blood sampling, which is a commonly used site for post-mortem analysis, is less subject to post-mortem redistribution than central blood sampling [17]. The presence of co-intoxicants can represent another confounder, and our pilot study has shown that co-intoxicants with a CNS depressant (mainly benzodiazepines and alcohol) or a CNS stimulant (mainly cocaine) are present in 33.6% and 30.3% of cases, respectively. The presence of CNS depressants can lead to an additive pharmacological effect with respiratory depression and sedation occurring at lower opioid doses [47], while the presence of cocaine, specifically in chronic administration, can lead to a rapid clearance of opioids, mainly methadone [48]. This demonstrates that the interplay of several factors such as genetic polymorphisms and co-intoxications may affect opioid concentrations in post-mortem samples. The presence of co-medication can present another complexity. The metabolic activity of a drug-metabolizing enzyme is not only modulated by genetics but also by the presence of co-medications. Co-medications which are inducers or inhibitors of a drug-metabolizing enzyme can alter the genotype-predicted phenotype of the enzyme, a process known as phenoconversion. For example, if an individual carrying a genotype-predicted *CYP2D6* normal metabolizer (NM) phenotype is administered a strong CYP2D6 inhibitor, the patient’s *CYP2D6* genotype-predicted phenotype will likely be converted to a poor metabolizer [49]. A study by Lam et al. 2014, demonstrated that the presence of a strong CYP2D6 inhibitor (i.e., antidepressants including paroxetine or fluoxetine) had a large influence on the concentrations of codeine metabolites and there were wide variations in the morphine/codeine ratios in post-mortem samples that were not explained by the *CYP2D6* genotype alone [16,24]. Finally, the frequencies of gene variants in cytochrome P450 genes and other genes involved in the metabolism and response of opioids vary greatly across populations of different ancestries. For example, the frequency of the 118G-allele is reported to be 1% in Africans, 16% in Europeans, but 42% in South Asians (www.ensembl.org). Therefore, information on the ancestry of the deceased must be considered when associations to show allele enrichment between post-mortem cases and control populations are conducted.

To date, genetic investigation is not routinely used in sudden or accidental opioid deaths, which can lead to inaccurate determinations of the cause of death. Pharmacogenetics may hold promise in the field of forensic toxicology. Gene variants in drug-metabolizing enzymes, drug transporters and drug receptors can alter the therapeutic/toxic doses of opioids and the opioid-receptor sensitivity, thus, resulting in fatal opioid toxicity. Therefore, pharmacogenetic analysis should be considered in unintentional deaths associated with opioids. Furthermore, the documented efficacy of genome-wide approaches for predicting opioid concentrations warrants a shift from single-gene approaches to capture the polygenic nature of opioid toxicity. In addition to pharmacogenetics, a complete and thorough post-mortem toxicological investigation should be conducted, including the identification of co-intoxications, co-medications, complete medical and demographic history and site of sample collection. Once limitations are overcome, findings validated and multigene panels made available to identify subjects at risk, there is hope and promise that the number of accidental overdoses can be decreased as research progresses.

## 5. Conclusions

Opioid-related mortality is a worldwide concern. Here, we systematically analyzed published literature, including results from our pilot study, on the relevance of using pharmacogenetics to determine the cause of accidental opioid toxicity using post-mortem samples. Our present analysis highlights two important findings: (1) genetic variants in opioid-metabolizing enzymes, opioid transporters and opioid receptors can be analyzed in post-mortem blood samples, and (2) genetic variation in drug-metabolizing enzymes, mainly *CYP2D6*, *CYP2B6* and *CYP3A4*, showed a significant correlation with the parent-opioid-to-metabolite ratios and may serve as an adjunct in certifying accidental opioid toxicity.

## Figures and Tables

**Table 1:** Characteristics of post-mortem studies included in this brief review (*n* = 18).

Study	Study Design	N	Ethnicity/ Ancestry	% Male		Age, Mean (sd), Years	Phenotype	Opioid	Gene	Variant	Findings	Co-Intoxicants Assessed
Jannetto et al., 2002 [17]	Case– control ^1^	Cases, *n* = 15 Controls, *n* = 26	Caucasian, *n* = 13 African-American, *n* = 2	53.3		39.9 (9.03)	Variant frequency, opioid blood concentration	Oxycodone	*CYP2D6*	PM, IM, NM and UM	There was no statistical difference in the post-mortem oxycodone concentrations between NM, IM, or PM ^5^ groups, (*p* > 0.05). The prevalence of PMs and IMs combined was not significantly higher than the control group, (*p* > 0.05). However, genotyping of CYP2D6 may serve as a molecular autopsy for certifying oxycodone mortality.	BZDs, ADs, alcohol and other drugs were present in some of the cases.
Levo et al., 2003 [19]	Retrospective cohort	33	European	33.3		65	Opioid and metabolite blood concentration	Tramadol	*CYP2D6*	PM, IM, NM and UM	There was a decrease in the tramadol/O-desmethyltramadol metabolite ratio when the number of functional alleles increased.	BZDs and ADs were present in some of the cases.
Jin et al., 2005 [18]	Retrospective cohort	25	Caucasian, *n* = 22 African-American, *n* = 1 Native-American, *n* = 2	36		48 (17.6)	Opioid and metabolite blood concentration	Fentanyl	*CYP3A4* *CYP3A5*	*1B *3	The average fentanyl concentration and the norfentanyl/fentanyl ratio of CYP3A4*1B wild type and CYP3A5*3 homozygous variant cases were higher than those of the CYP3A4*IB variant cases, but not statistically significant. Genotyping of CYP3A4*1B and CYP3A5*3 genes may serve as an adjunct in certifying fentanyl toxicity.	BZDs, ADs, alcohol and other drugs were present in some of the cases.
Buchard et al., 2010 [20]	Retrospective cohort	90	European	76.7		-	Opioid and metabolite blood concentration	Methadone	*ABCB1*	rs1045642 rs2032582 rs1128503	No significant associations between ABCB1 variants and post-mortem methadone and metabolite concentrations.	BZD, alcohol and other drugs were present in some of the cases.
Bunten et al., 2010 [21,22]	Retrospective cohort	40	Caucasian	85		31 (1.67)	Opioid blood concentration	Methadone	*CYP2B6*	rs2279343 (A785G, *4) rs3745274 (G516T, *9) (G516T and A785AG, *6)	CYP2B6 *4, *9, and *6 alleles were found to be associated with higher post-mortem methadone concentrations in blood (*p* ≤ 0.05).	BZD, alcohol and other drugs were present in some of the cases.
									*OPRM1*	A118G	The A118G variant was not associated with higher post-mortem methadone concentrations (*p* = 0.39). The A118G variant was associated with benzodiazepine concentration when it was combined with methadone. Heterozygous individuals demonstrated a 2.4-fold higher mean benzodiazepine concentration as compared with homozygous wild-type carriers, (*p* = 0.004).	
Bunten et al., 2011 [23]	Case–control ^1^	Cases, *n* = 84 Controls, *n* = 100	Caucasian	73.8		33.2 (1.12)	Variant frequency	Methadone	*CYP2B6*	rs2279343 (A785G, *4) rs3745274 (G516T, *9) (G516T and A785AG, *6)	The G516T and A785G variants were higher in the post-mortem population than in the control group. However, this difference was not statistically significant.	-
									*OPRM1*	A118G	The prevalence of the OPRM1 118G variation was significantly higher in the control population (*p* = 0.0046), which might indicate a protective mechanism against opioid toxicity.	
Frost et al., 2012 [16]	Retrospective cohort	34	-	50		49.9 (14.4)	Opioid and metabolite blood concentration	Codeine	*CYP2D6*	PM, IM and EM	There is a large variability in the morphine/codeine ratio after codeine intake in forensic autopsy cases. Morphine levels cannot be predicted from codeine concentrations. CYP2D6 genotyping may be of interest in cases with unexpectedly high or low M/C ratios.	BZD, AD, alcohol and other drugs were present in some of the cases.
Lam et al., 2014 [24]	Case–control ^2^	Cases, *n* = 36, Controls, *n* = 32	-	50		49	Opioid and metabolite blood concentration	Codeine	*CYP2D6*	PM, IM and EM	The morphine-to-codeine ratio was significantly correlated with the presence of a CYP2D6 inhibitor at varying potencies (*p* = 0.0011). CYP2D6 genotype was not significantly associated with morphine/codeine ratio (*p* = 0.20).	BZDs, ADs, alcohol, opioids and other drugs were present in the cases.
									*ABCB1*	rs1128503 (C1236T) rs2032582 (G2677T/A) rs1045642 (C3435T)	Individuals who carried the 1236T variant had statistically lower morphine concentrations than wild-type carriers (*p* = 0.004).	
									*OPRM1* *COMT* *UGT2B7*		No significant association between variants and opioid concentrations.	
Richards- Waugh et al., 2014 [25]	Case–control ^3^	Cases, *n* = 238 Controls, *n* = 258	Caucasian	-		-	Variant frequency, opioid and metabolite blood concentration	Methadone	*CYP3A4*	rs2246709 rs3735451 rs4646437 rs2242480 rs4987161 rs4986910 rs2740574 (*1B)	SNPs rs2242480 and rs2740574 (*1B) were enriched within the methadone-only overdose fatalities compared with the control group and the general population. There was no statistical difference in either methadone concentrations or methadone/EDDP ratios for all SNPs.	Only BZDs were present in selected cases.
Christoffersen et al., 2016 [26]	Case–control ^4^	Cases, *n* = 274 Control, *n* = 309	Caucasian	79		41	Variant frequency	Methadone, Morphine	*ABCB1*	rs1045642 rs2032582 rs1128503 rs9282564 rs2235036	There was a significantly lower frequency of the AG and GG genotypes in the rs9282564 in deceased patients with opioid addiction compared with living patients with opioid addiction, (*p* = 0.027).	-
									*COMT*	rs4680 rs4633 rs4818	There was a significantly lower frequency of the AA genotype in the rs4680 (Val158Met) variant in deceased patients with opioid addiction compared with living patients with opioid addiction, (*p* = 0.0028).	
									*OPRM1* *UGT2B7* *CYP3A5* *CYP2B6* *CYP2D6*	Several variants tested	No significant associations were detected.	
Fonseca et al., 2016 [27]	Retrospective cohort	100	European	56	65		Opioid and metabolite blood concentration	Tramadol	*CYP2D6*	PM, IM, EM and UM	The metabolism of tramadol is correlated with the phenotype of the metabolizer.	BZDs, ADs, alcohol, opioids and other drugs were present in the cases.
Frost et al., 2016 [28]	Retrospective cohort	23	-	56.6	47.3 (12.0)		Opioid and metabolite blood concentration	Codeine	*CYP2D6*	PM, IM, EM and UM	There was a large variability in calculated ratios of codeine metabolites to codeine, and CYP2D6 genotype was not a reliable predictor of these ratios.	BZDs, ADs, alcohol, opioids and other drugs were present in the cases.
Ahmad et al., 2017 [29]	Case–control ^3^	Cases, *n* = 125 Controls, *n* = 255	Caucasian	-	-		Variant frequency, opioid blood concentration	Methadone	*CYP2B6*	*1, *2, *5, *8, *9, *15 rs2279344 rs4803419 rs8192719	SNPs rs3745274 (*9) and rs8192719 exhibited significant differences in the methadone-only group compared to the control group. For these two SNPs, the minor allele frequency in the methadone-only cases was greater than that of the control group. Higher blood methadone concentrations were observed in individuals who were genotyped homozygous for SNP rs3211371 (*5).	No co-intoxicants present in cases.
Wendt et al., 2019 [30,31]	Retrospective cohort	208	European	61.4	56.1		Opioid and metabolite blood concentration	Tramadol	*CYP2D6* *ABCB1* *UGT2B7* *OPRM1* *COMT*	-	The UGT2B7 is a potentially significant explanatory marker for tramadol/O-desmethyltramadol variability. A set of 16 loci from 5 genes can predict metabolizer phenotype with over 90% accuracy, which is greater than using CYP2D6 alone.	BZDs, ADs, alcohol, opioids and other drugs were present in the cases.
Boyle and Stock 2020 [32]	Retrospective cohort	75	Caucasian	97	51		Variant frequency	Hydrocodone Oxycodone Tramadol	*CYP2D6*	PM, IM, EM and UM	The UM phenotype is not over-represented in opioid overdose deaths.	BZDs, ADs, alcohol, opioids and other drugs were present in some of the cases.
Wendt et al., 2020 [33]	Retrospective cohort	37	European	64.9	52.3 (19.0)		Opioid and metabolite blood concentration	Tramadol	*GWAS*	-	Five SNPs were found to be associated with decreased O-desmethyltramadol/tramadol ratio, including rs9384825, rs62435418, rs72732317, 184199168, and rs79983226.	BZDs, ADs, alcohol, opioids and other drugs were present in the cases.
Iwersen-Bergmann et al., 2021 [34]	Retrospective cohort	107	-	73.8	41		Opioid blood and brain concentration	Methadone	*ABCB1*	rs1045642 rs2032582 rs1128503	For SNP rs1045642, the methadone medulla/blood ratios of the T/T genotype were significantly higher than those of the other genotypes (T/T vs. T/C, *p* = 0.002; T/T vs. C/C *p* = 0.004).	BZDs, alcohol, opioids and other drugs were present in the cases.
Jakobsson et al., 2021 [35]	Retrospective cohort	174	Caucasian	64	57 (14.7)		Opioid and metabolite blood concentration	Oxycodone	*CYP2D6*	PM, IM, EM and UMs	PMs and IMs had significantly higher oxycodone and noroxycodone concentrations compared to EMs and UMs. CYP2D6 phenotype was equally distributed between cause of death groups (accidental, suicide, undetermined, natural). The concentration ratio between oxymorphone and oxycodone depended on the CYP2D6 activity when death was unrelated to intoxication.	BZDs, ADs, alcohol, opioids and other drugs were present in selected cases.

^1^ Controls were healthy volunteers; ^2^ Cases were accidental deaths and controls were deaths caused by suicide; ^3^ Controls were deaths not caused by opioids or other intoxicants.^4^ Controls were living patients with opioid addiction; ^5^ PM = *3/*3, *4/*4, *5/*5, *3/*4, *4/*5; IM = *1/*4, *1/*3, *1/*5; NM = *1/*1. Average age is reported for cases only in case–control studies. AD = antidepressants; BZD = benzodiazepines; COMT = catechol-o-methyltransferase; EDDP = 2-ethylidene-1,5-dimethyl-3,3-diphenylpyrrolidine; GWAS = genome-wide association study; OPRM1 = mu-opioid receptor encoding gene; UGT = UDP-glycosyltransferase; PM = poor metabolizer; IM = intermediate metabolizer; UM = ultrarapid metabolizer; NM = normal metabolizer; EM = extensive metabolizer.

**Table 2:** Sample demographics and clinical information.

	Overall (N = 119)
Sex	
Female	24 (20.2%)
Male	78 (65.5%)
Age	
Mean (SD)	42.7 (11.5)
Median (Min, Max)	41.0 (16.0, 71.0)
Opioid	
Fentanyl	64 (53.8%)
Methadone	41 (34.5%)
Carfentanil	5 (4.2%)
Morphine	2 (1.7%)
Hydromorphone	2 (1.7%)
Fentanyl and methadone	2 (1.7%)
Fentanyl and hydromorphone	1 (0.8%)
Oxycodone	1 (0.8%)
Heroin	1 (0.8%)
CNS stimulants	
Cocaine	36 (30.3%)
Methamphetamine	30 (25.2%)
Amphetamine	19 (16.0%)
Benzoylecgonine	9 (7.6%)
MDMA	4 (3.4%)
Benzodiazepine	
Etizolam	5 (4.2%)
Diazepam	1 (0.8%)
Diazepam and nordiazepam	2 (1.7%)
lorazepam	1 (0.8%)
Bromazolam	1 (0.8%)
Alcohols	
Ethanol	30 (25.2%)
Antidepressants	
Citalopram	4 (3.4%)
Mirtazapine	1 (0.8%)
Paroxetine	1 (0.8%)
Cannabis	
THC	1 (0.8%)
Others	
Pseudoephedrine	4 (3.4%)
Dextromethorphan	2 (1.7%)
Pheniramine	1 (0.8%)
Antipsychotics	
Olanzapine	1 (0.8%)

**Table 3:** Observed genotype and minor allele frequency in methadone-related fatalities (*n* = 41).

	Overall	Females	Males	*p*-Value ^a^	MAF
CYP2B6*4				0.098	0.3
1 1	10 (43.5%)	3 (60.0%)	7 (41.2%)		
1 4	12 (52.1%)	1 (20.0%)	10 (58.8%)		
4 4	1 (4.3%)	1 (20%)	0 (0%)		
CYP2B6*9					
1 1	11 (44.0%)	5 (83.3%)	6 (33.3%)	0.007 *	0.3
1 9	13 (52.0%)	0 (0%)	12 (66.7%)		
9 9	1 (4.0%)	1 (16.7%)	0 (0%)		
*OPRM1* (A118G)					
A A	37 (90.2%)	5 (83.3%)	18 (100%)	0.25	0.05
A G	4 (9.8%)	1 (16.7%)	0 (0%)		

^a^ Fisher’s exact test for categorical variables. * post-hoc pairwise Fisher test was conducted, the *1/*1 and *1/*9 genotypic groups were significantly different among males and females, *p*-value after false-discovery rate correction (fdr) is 0.04. MAF = minor allele frequency.

## Data Availability

The data that support the findings of this study are not publicly available because they contain genetic information that could compromise the privacy of research participants but are available from the corresponding author, D.J.M., upon reasonable request.
